# Structure and function of MDM2 and MDM4 in health and disease

**DOI:** 10.1042/BCJ20240757

**Published:** 2025-02-17

**Authors:** Ivy Yiyi Zhu, Alec Lloyd, William R. Critchley, Queen Saikia, Dhananjay Jade, Aysha Divan, Elton Zeqiraj, Michael A. Harrison, Christopher J. Brown, Sreenivasan Ponnambalam

**Affiliations:** 1 School of Molecular and Cellular Biology, University of Leeds, Leeds LS2 9JT, U.K; 2 School of Biomedical Sciences, University of Leeds, Leeds LS2 9JT, U.K; 3 Institute of Molecular and Cell Biology (IMCB), Agency for Science, Technology and Research (A*STAR), Singapore

**Keywords:** MDM2, MDM4, E3 ubiquitin ligase, p53, 26S proteasome, cancer

## Abstract

Both mouse double-minute 2 (MDM2), an E3 ubiquitin ligase, and its closely related paralog, MDM4, which lacks E3 activity, play central roles in cellular homeostasis. MDM-linked dysfunction is associated with an increased risk of oncogenesis, primarily through targeting the tumor suppressor protein p53 for ubiquitination and degradation. Recent studies have revealed multifaceted roles of MDM proteins that are p53 independent with implications for their oncogenic properties. This review aims to provide an overview of MDM2 and MDM4, by assessing gene and protein structure and implications for protein–protein interactions and functions in cell and animal physiology. We also explore MDM2 and MDM4 role(s) in angiogenesis, a critical feature of solid tumor growth and progression. Finally, we discuss the current landscape in the development of MDM2 and MDM4 inhibitors for cancer therapy.

## Introduction

Mouse double-minute 2 (MDM2) and its paralog MDM4 (also termed MDMX) were originally identified as mouse oncoproteins that bind to the tumor suppressor protein p53 [1,2]. Several lines of research revealed that conserved human orthologs, also known as hMDM2 (HDM2) and hMDM4 (HDM4), are widely expressed across most human tissues. MDM proteins belong to the E3 ubiquitin ligase superfamily; MDM proteins belong to the E3 subfamily characterized by the Really Interesting New Gene (RING) domain (~50 residues). These enzymes play critical roles in the ubiquitination of various substrates, regulating their activity, localization and degradation, thus regulating multiple cellular processes [3]. MDM gene conservation in metazoan species has facilitated comparison of human and murine MDM function in health and disease outcomes. Subsequent studies have implicated MDM functionality as oncoproteins that are frequently overexpressed in a range of human cancers, thus promoting tumorigenesis [4].

**Figure 1:**
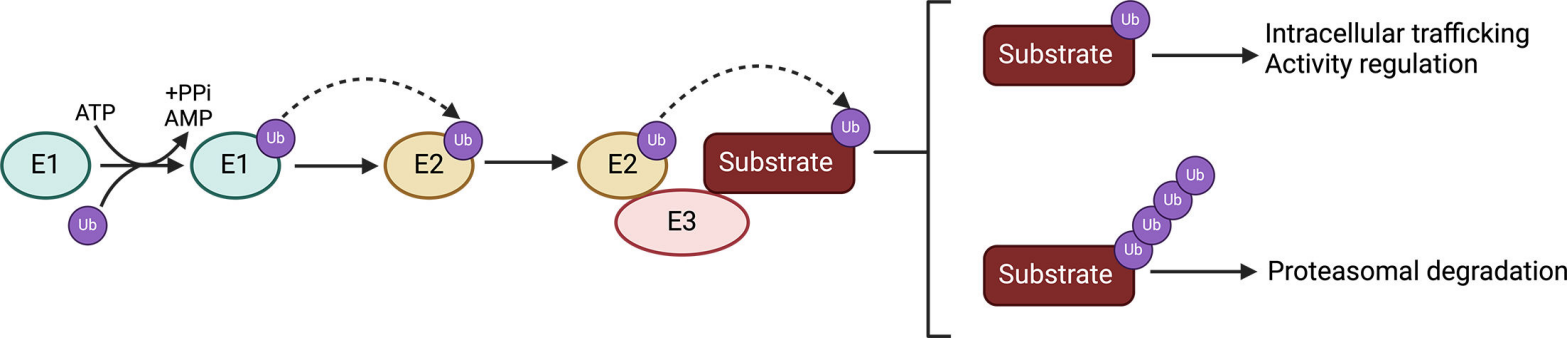
Ubiquitination pathway overview. Schematic representation of the ubiquitination pathway involving E1 ubiquitin-activating, E2 ubiquitin-conjugating and E3 ubiquitin-ligase enzymes. Ubiquitin is activated by an E1 enzyme in an ATP-dependent manner before being transferred to an E2 enzyme. Then, the ubiquitin is transferred directly onto the substrate, catalyzed by RING-type E3 ubiquitin ligase; or indirectly by other types of E3 enzymes (not shown). Ubiquitination can lead to monoubiquitination, the attachment of a single ubiquitin molecule or polyubiquitination, where a chain of ubiquitin molecules is formed (created with BioRender.com). Abbreviation: RING, Really Interesting New Gene.

The most well-studied cancer-linked role of MDM proteins is their ability to bind, ubiquitinate and target p53 for degradation by the 26S proteasome [4]. Ubiquitination is one type of post-translational modification facilitated by an E1-E2-E3 enzyme cascade, which mediates the attachment of ubiquitin to the ε-amino group on the side chain of lysine residues on target or client proteins [5]. Ubiquitin conjugation can be extended into a linear or branched structure termed polyubiquitination; a minimum of four linked ubiquitin units are required for 26S proteasomal degradation of target substrates (Figure 1) [6]. Conversely, monoubiquitination involves attaching a single ubiquitin molecule to the target, which usually modulates protein localization and activity [7]. Tumor suppressor p53 levels are usually low under physiological conditions: MDM activity promotes ubiquitination and subsequent degradation [3]. In response to cellular stresses such as DNA damage, oxidative stress and oxygen deprivation, MDM proteins are inhibited and p53 levels increase, leading to activation of multiple signaling pathways contributing to cell cycle arrest, DNA repair and apoptosis [3]. It is thus unsurprising that p53 is one of the most frequently mutated oncoproteins in human cancers, with >50% cancers displaying mutations in one or both *TP53* alleles; such mutations can cause loss or gain of function in p53 activity that contributes to tumorigenesis [8]. In addition, frequent MDM gene amplification and overexpression in wild-type (wt) *TP53* patients represses p53-dependent tumor suppression, making these gene products highly relevant to anti-cancer strategies [4]. Furthermore, considerable evidence now supports p53-independent activities of MDM proteins in a range of cellular processes, such as cell cycle control, cell differentiation and metabolism, further expanding proto-oncoprotein functionality [3]. This review discusses the structure and function of MDM2 and MDM4 oncoproteins in terms of regulation of p53. p53-independent MDM function will also be considered. Finally, protein–protein interactions (PPIs) between MDM gene products and p53 may influence angiogenesis, a critical feature of tumor growth and metastasis.

## Genetics of MDM2 and MDM4

### MDM gene structure

**Figure 2:**
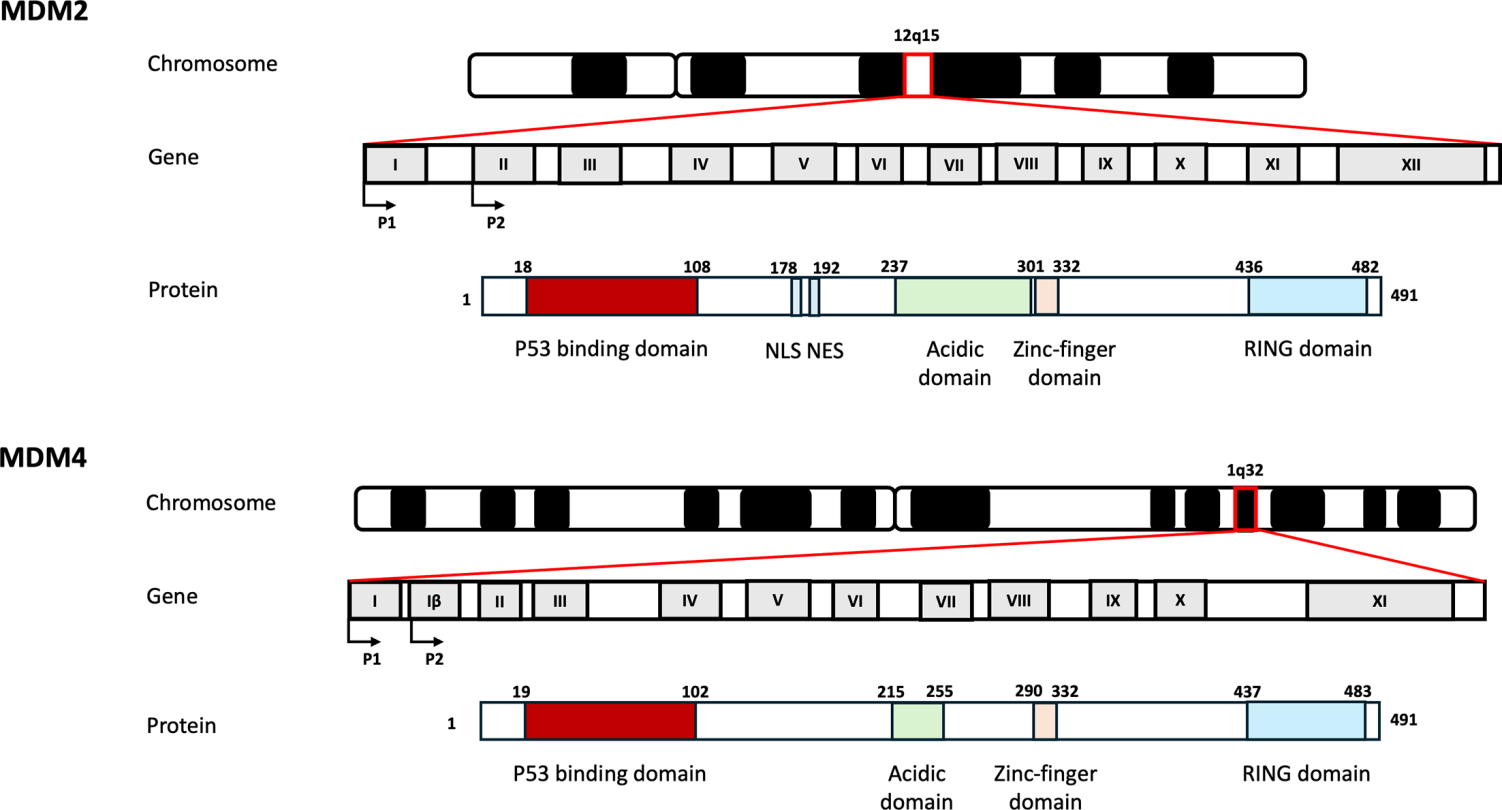
*MDM2* and *MDM4* genes and proteins. Graphical representation of the chromosomal location, gene structure with exon organization, and protein structures of MDM2 and MDM4. The two promoters of each gene are shown as arrows. Abbreviations: NES, nuclear export signal; NLS:, nuclear localization signal; RING, Really Interesting New Gene.;NES: nuclear export signal.

*MDM2* and *MDM4* gene loci are located on human chromosomes 12q15 and 1q32, respectively. These genes exhibit high sequence similarity (>80%) and are believed to have arisen due a gene duplication event ~440 million years ago during vertebrate evolution [9]. *MDM2* and *MDM4* exhibit high structural resemblance, encoding proteins with ~32% identity and ~90% similarity. *MDM2* contains 12 exons, whereas *MDM4* has 11 exons; *MDM2* encodes at least 11 protein isoforms, whereas *MDM4* encodes at least five isoforms (Figure 2) [10]. Both genes contain two promoters: the P1 promoter regulates basal gene transcription, and the P2 promoter is activated under cellular stress conditions [11]. The P1 promoter of *MDM2* is located upstream of the first exon and mediates transcription from the first exon but skips the second exon, producing both the full-length P90 protein isoform and a shorter P76 isoform [12]. The P90 isoform is translated from exon 3 and is capable of binding and inhibiting p53, whereas P76 is translated from exon 4, lacks the N-terminal p53-binding region and acts as an inhibitor of P90, thus promoting p53 activity [13]. Additionally, P76 is involved in the ubiquitination and degradation of MDM4, adding further complexity to its activity in p53 regulation [14]. The P2 promoter, found within the first intron and transcribed from exon 2, preferentially gives rise to the P90 isoform more efficiently than P1-mediated gene transcription [11]. The P2 promoter is known to be activated by p53 binding with the presence of two p53 response elements, forming part of a negative feedback loop to modulate p53 levels [15]. Notably, transcription from the P1 promoter can also be modulated by regulatory factors such as phosphatase and tensin homolog (PTEN), and the P2 promoter can be regulated by p53-independent factors such as the Ets family of transcription factors [16]. Intriguingly, an additional P3 promoter located within intron 3 has been documented and repressed by p53 binding but has not been widely studied [17].

Similar to *MDM2*, the P1 promoter within *MDM4* is located upstream of the first exon, while the P2 promoter is located within a novel exon termed 1β [11]. Transcription initiated from the P2 promoter produces an MDM4 protein isoform termed MDM4-L, which is 18 amino acids longer than the major MDM4 isoform; MDM4-L lacks the ability to inhibit p53-dependent transcription [11]. Nevertheless, MDM4-L retained the ability to promote MDM2-mediated p53 ubiquitination, while also promoting MDM2 autoubiquitination [11].

Recent studies identified GC-rich sequences within the MDM promoters that form G-quadruplex DNA arrangements: these inhibit *MDM2* but promote *MDM4* gene transcription [18]. Multiple proteins, mainly helicases, have been shown to interact with G-quadruplexes to regulate its folding [19]. Furthermore, targeting the G-quadruplex using small compound ligands has been shown to affect MDM protein expression [18,19]. Such genetic regulation represents parts of a regulatory pathway in controlling MDM expression that could be potentially targeted in disease states.

### MDM alternative RNA splicing

**Figure 3:**
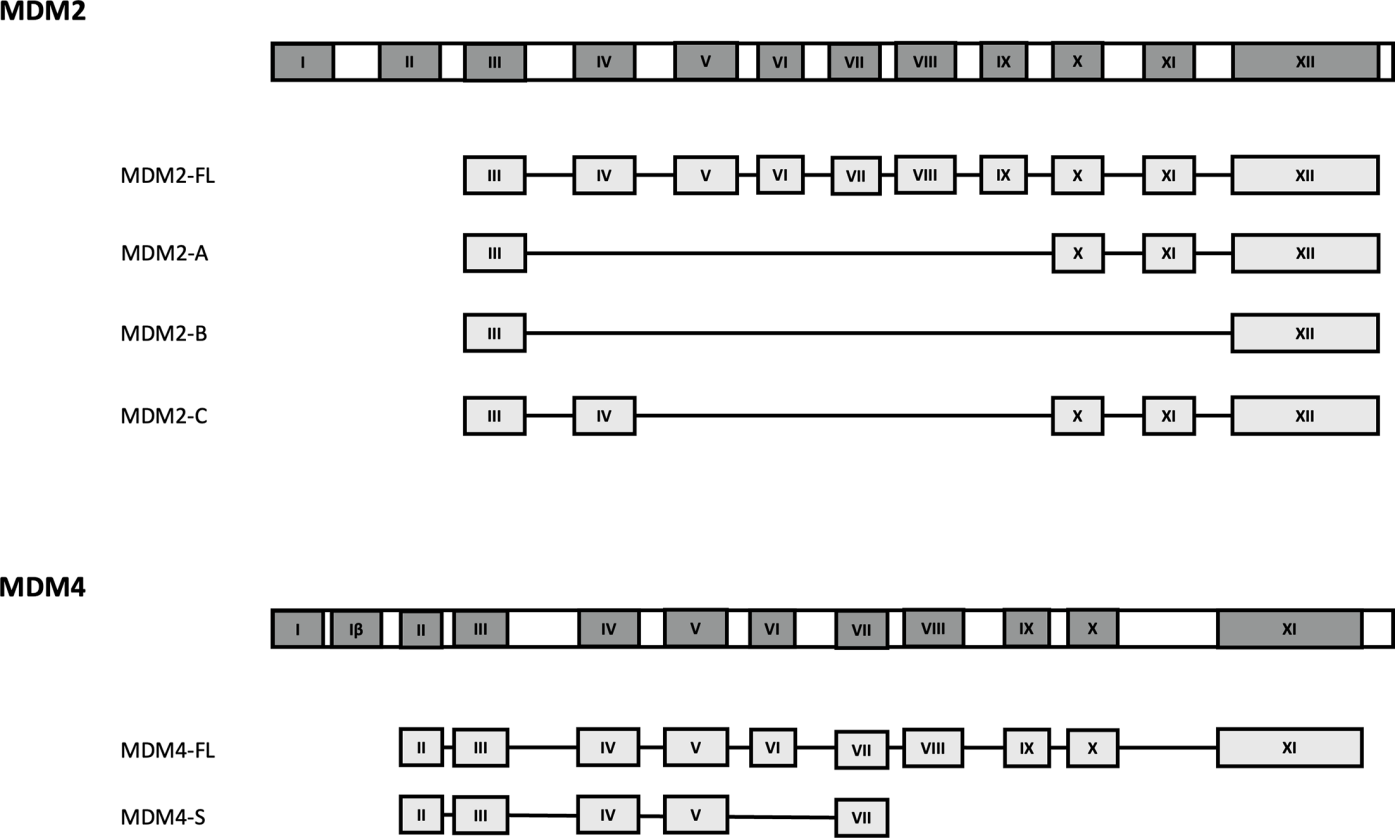
MDM2 and MDM4 protein isoforms. MDM mRNA splice variants encoding protein isoforms are depicted underneath *MDM2* and *MDM4* genes. Numbers indicate the different exons within the *MDM2* and *MDM4* genes. MDM2-FL:, full-length MDM2; MDM4-FL:, full-length MDM4.

Alternative splicing of *MDM2* and *MDM4* primary RNA transcripts can generate multiple mRNA transcripts. To date, 72 *MDM2* and 8 *MDM4* mRNA splice variants have been identified; some of these are highly expressed in cancers and associated with tumor growth *in vivo* [20–22]. However, only some of these mRNA splice variants have been confirmed as protein isoforms. MDM2-A, MDM2-B and MDM2-C are the most frequently detected and studied MDM2 protein isoforms in human cancer (Figure 3) [21]. These isoforms lack p53-binding sites yet retain the ability to bind to full-length MDM2 (MDM2-FL) through their carboxy-proximal RING domains [21]. Furthermore, MDM2-A and MDM2-B retain both auto-ubiquitination and p53 ubiquitination activities and can form heterodimers with MDM2 or MDM4, indicating a functional regulatory role [23]. Less work has been carried out on MDM2-C, where its ubiquitination activity has only been demonstrated *in vitro* [24]. Interestingly, despite the lack of p53 binding, MDM heterodimers containing MDM2-A or MDM2-B and MDM4 exhibit higher p53 ubiquitination activity than MDM2-FL:MDM4 heterodimers [23]. MDM2-A lacks exons 4–9: this isoform is commonly detected in rhabdomyosarcomas and acts as an activator for p53-dependent growth arrest *in vitro*; however, this effect is not observed *in vivo* [22]. In addition, the role of MDM2-A in tumorigenesis seems to be context-dependent, whereas its overexpression in tumors did not increase the rate of tumorigenesis and is only found to promote cellular transformation in a *TP53*-null background *in vivo* [25,26]. MDM2-B lacks exons 4–11 and represents the most commonly found variant in human cancers [21]. MDM2-B is known for its role in promoting p53 activity by disrupting MDM2-FL interaction with p53 or promoting MDM2-FL phosphorylation, inhibiting its E3 ligase activity and contributing to the accumulation of mutant p53 [21,26,27]. MDM2-C lacks exons 5–9 and is predominantly associated with breast cancer [28,29]. MDM2-C does not inhibit p53 activity, despite colocalization with MDM2-FL [28]. Expression of MDM2-C *in vitro* in the absence of wt p53 promotes tumorigenesis, but its expression in breast cancer patients is correlated with a decreased mortality rate [28,29]. The activity of these MDM isoforms underscores the complex, isoform-specific role(s) of MDM2 gene products in cancer development and progression.

Fewer studies have been carried out on MDM4 isoforms, but MDM4-S is the most extensively characterized protein isoform. MDM4-S encodes the p53-binding domain with an additional 13 residues at the carboxyl terminus that is responsible for its increased p53 affinity (Figure 3) [30]. Although initially identified as a potent negative regulator of p53 and overexpressed in multiple cancers, subsequent work revealed that MDM4-S overexpression does not promote tumorigenesis *in vivo*, suggesting that increased MDM4-S levels are likely a consequence of tumorigenesis rather than a cause [31]. This discrepancy in function and outcome may be attributed to MDM4-S mRNA low translation efficiency and protein instability, thus reducing inhibitory activity against p53 [32,33]. Other variants such as MDM4-A and MDM4-211 have also been associated with tumor development and progression, but the exact role(s) remain incompletely understood. MDM4-A, which lacks most of the acidic domain due to the exclusion of exon 9, has not been detected at protein level. Nevertheless, it is the most commonly expressed isoform in melanomas and is associated with a lower survival rate in patients [34]. Functional studies in cancer cell lines indicate that MDM4-A retains the ability to bind and inhibit p53-mediated transcriptional activation, potentially accounting for its correlation with reduced survival in melanoma patients [35]. In contrast, MDM4-211 is characterized by the exclusion of exons 3–10 and part of exon 11, resulting in the loss of p53-binding ability [36]. However, MDM4-211 stabilizes the interaction between full-length MDM2 and p53 by binding to MDM2 through RING domain interactions, thereby preventing the proteasomal degradation of both proteins [36]. Despite this stabilizing effect on p53, MDM4-211 suppresses its tumor-suppressive function by promoting its association with MDM2 [36].

## MDM protein structure and function

### N-terminal domain

Full-length MDM2 and MDM4 proteins have highly conserved domains and organization, including an N-terminal p53-binding domain, a central acidic and zinc finger domain, and a C-terminal RING domain. The N-terminal domain is primarily composed of α-helices and serves as the major p53-binding site; this region is also capable of interacting with other proteins such as insulin-like growth factor 1 receptor (IGF-1R), a membrane protein and receptor tyrosine kinase (Table 1) [47]. This MDM N-terminal domain contains an unstructured region (amino acids 1–24), which is believed to act as a flexible lid with autoinhibitory activities that blocks the major p53 interaction site [48]. Although the regulatory mechanism that switches this lid between open and closed states remains elusive, computational studies have suggested that phosphorylation at several sites within this region influences the binding of MDM2 to p53 [48–50]. While numerous studies have focused on the S17 phosphorylation site in the MDM2 N-terminus, evidence shows that changes at this site have minimal impact on its binding to p53 [48]. This suggests that alternative or multiple phosphorylation sites may act co-operatively to influence the MDM lid conformation and functionality, highlighting the complexity of this regulatory mechanism. Furthermore, this unstructured region with proposed ‘lid’ function contributes to the stabilization of p53 interactions and forms structured helices upon binding to small molecule inhibitors [51,52]. Recent studies have identified residue I19 as a crucial residue in ordering this lid region [53]. Similarly, the highly similar N-terminal domain of MDM4 also has ‘lid’ capability that affects p53 interactions in a phosphorylation-dependent manner [54]. These insights underscore the critical role of the N-terminal MDM lid region in ligand binding; this region is a major target for modulating PPIs with implications for cellular responses in health and disease.

**Table 1:** Summary of MDM2 and MDM4 protein–protein interactions (PPIs).

Category	Partner	Interaction type	Role of MDM proteins	Ref
Membrane proteins/receptors	IGF-1R	Negative regulation	MDM2 mediates the ubiquitination and degradation of IGF-1R	[37]
	AR	Negative regulation	MDM2 mediates the ubiquitination and degradation of AR	[38]
	Notch1	Positive regulation	MDM2 ubiquitinates and activates Notch1	[39]
Signaling effectors	PKCβ	Positive regulation	MDM2 ubiquitinates and activates PKCβ	[40]
	pVHL	Negative regulation	MDM2 mediates the ubiquitination and degradation of pVHL	[41]
	CHK2	Negative regulation	MDM2 mediates the ubiquitination and degradation of CHK2	[42]
Transcription modulators	p53	Negative regulation	MDM2/MDM4 directly inhibit, ubiquitinate and degrade p53	[4]
	E2F1	Positive regulation	MDM2 binds E2F1, inhibiting its ubiquitination by SCF^skp2^ and promotes its stabilization	[43]
Others	CDH1	Negative regulation	MDM2 mediates the ubiquitination and degradation of CDH1	[44]
	Nbs1	Negative regulation	MDM2 directly inhibits Nbs1	[45]
	BCL2	Positive regulation	MDM4 bind BCL2, enhancing its interaction with p53 and promotes apoptosis	[46]

IGF-1R , Insulin-like growth factor 1 receptor. AR , androgen receptor. PKCβ , Protein kinase Cβ. pVHL , von Hippel-Lindau. CHK2 , checkpoint kinase 2. E2F1 , E2 transcription factor 1. CDH1 , E-cadherin.

### Central acidic and zinc finger domain

The central MDM region contains an acid-rich and zinc finger domains. The MDM acidic domain is less conserved and was initially thought to play a minor role in protein function. However, emerging evidence suggests a critical role(s) in PPIs, ubiquitination and homodimer formation [55]. This acid-rich MDM2 domain facilitates p53 degradation through both ubiquitination-dependent and ubiquitination-independent pathways, with the latter pathway involving interaction with the 26S proteasome [6,56]. Similarly, the acidic MDM4 domain also inhibits p53, whereas mutation of either the dual tryptophan (WW) or tryptophan-phenylalanine (WF) motifs can enhance p53 binding to DNA [57]. Recent study identified an additional layer of function in such MDM motifs: they have auto-inhibitory properties through binding to the N-terminal MDM region, thus blocking p53 binding [57,58]. The MDM zinc finger domain contributes to the stabilization of MDM2-MDM4 complex and suppresses p53 transactivation activity upon phosphorylation [59]. The zinc finger interacts with regulatory proteins such as ribosomal proteins L5 and L11; mutations within the zinc finger disrupt such interactions, leading to reduced L11-mediated inhibition of MDM2 and enhanced p53 degradation [60,61]. Additionally, mutation in the MDM2 zinc finger domain prevents nuclear export, thus promoting p53 ubiquitination [61].

### C-terminal RING domain

The C-terminal RING domain of MDM proteins is conserved and characterized by a consensus sequence that includes two zinc ion binding pockets required for functionality [62]. The RING domain harbors two key functions: facilitating PPI and catalyzing ubiquitination of protein substrates such as p53 [62]. How the RING domain carries out this latter catalytic regulation is relatively poorly understood. One possibility that bringing a specific E2 enzyme into close proximity to the target or client substrate is sufficient; alternatively, a more complex catalytic mechanism through which ubiquitin is mobilized onto a target site is possible. This RING domain is crucial for the formation of MDM2-MDM4 heterodimers while being dispensable for MDM2 homodimerization; MDM2-MDM2 complex formation instead relies on the extreme C-terminal residues distal to the RING domain [55]. Despite its high-sequence similarity, MDM4 lacks E3 ubiquitin ligase activity and is unable to form homodimers under physiological conditions [63]. Nonetheless, a recent study in single living cells expressing MDM4-EGFP (enhanced green fluorescent protein) found that MDM4 exists largely as homodimers [64]. However, no follow-up studies have been conducted to confirm this observation. The RING domain of MDM2 also promotes autoubiquitination within the complex with conflicting evidence on the exact regulatory mechanism [65]. Initial studies suggested that autoubiquitination leads to MDM2 degradation *in vitro*, but the same effect was not observed *in vivo* [65,66]. More recent findings indicate that polyubiquitinated MDM2 caused by autoubiquitination promotes increased p53 ubiquitination via increased recruitment of E2 ubiquitin-conjugating enzymes [67]. Further complexity in MDM protein activity and regulation is evidenced by the differential properties of MDM dimers; whereas MDM2 homodimers show higher autoubiquitination activity, MDM2-MDM4 heterodimers preferentially ubiquitinate p53 [62]. A recent study has identified a critical role for the MDM2-C449 residue in MDM2-MDM4 heterodimer formation [68]. Interestingly, mutation of MDM2-C449 does not affect p53 degradation, suggesting compensatory activity from MDM2 homodimers shifts from autoubiquitination to p53 ubiquitination when heterodimer formation is disrupted [68]. These findings highlight a complex regulatory network of MDM PPIs with multiple substrates.

### Other MDM protein features

MDM2 contains additional nuclear localization (NLS) and nuclear export (NES) signals located between the N-terminal and acidic domains; such features are absent from MDM4. These signals are important for nuclear p53 regulation by allowing MDM2 to shuttle between the nucleus and cytoplasm [21]. The presence and absence of NLS and NES explain the localization of MDM2 and MDM4 proteins, where MDM2 is primarily localized to the nucleus and MDM4 is mainly found in the cytoplasm [30]. Nonetheless, MDM4 can be transported into the nucleus via binding to MDM2 [69].

## MDM-regulated p53 binding and regulation

The oncoprotein-like activity of MDM2 and MDM4 mainly stems from their interaction with the ‘guardian of the genome’ and tumor suppressor, p53. Expression of p53 throughout the life cycle in all human cells and tissues indicates vital role(s) in DNA repair, cell cycle progression and cellular apoptosis [70]. Tight regulation of p53 levels by MDM proteins ensures low p53 levels under physiological conditions; however, p53 levels are elevated by cellular damage and stress to prevent propagation of cells with DNA damage [3].

### Direct inhibition of p53

**Figure 4:**
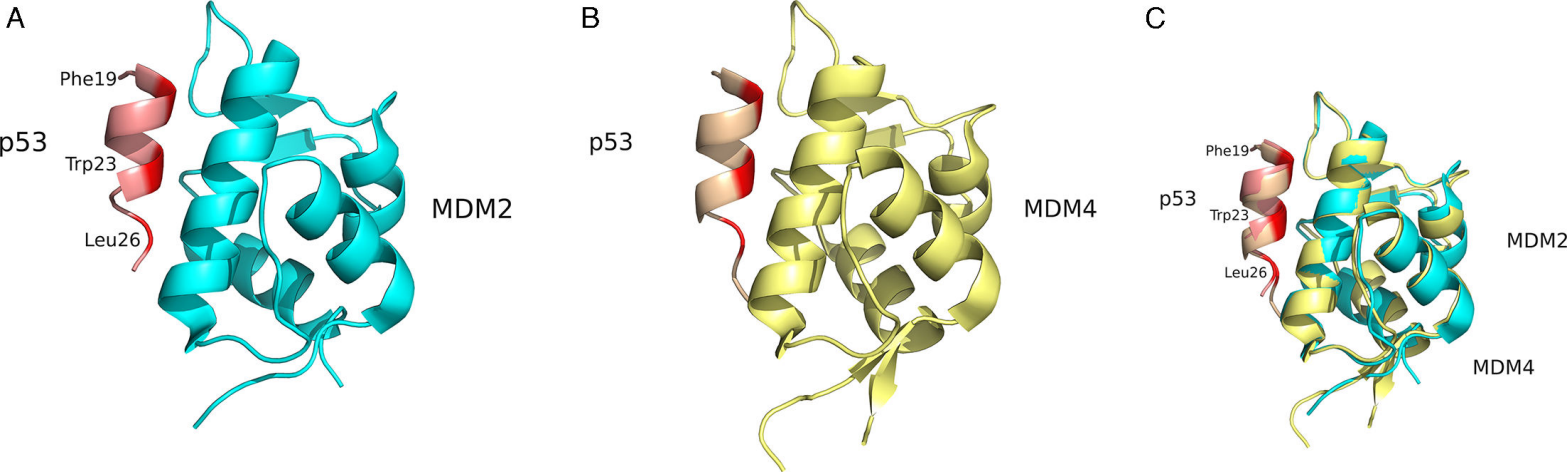
Structural models of MDM2-p53 interactions. **(**A**)** MDM2 (aquamarine) bound to the p53 peptide (pink) (PDB ID: 4HFZ)., **(**B**)** MDM4 (yellow) bound to the p53 peptide (sand) (PDB ID: 3DAB),. and **(**C**)** sSuperimposed structures. Key p53 interacting residues, Phe19, Trp23 and Leu26, isare shown and highlighted in red. Structures generated using PyMOL (www.pymol.org). MDM2, mouse double-minute 2; MDM4, mouse double-minute 4.

MDM proteins inhibit p53 levels and functionality through three major mechanisms. First, MDM2 and MDM4 directly inhibit the transcriptional activation of p53-responsive genes by binding to the p53 transactivation domain (TAD) through their N-terminal domain [71,72]. Second, MDM proteins dimerize and facilitate the proteasomal degradation of p53 via ubiquitination [64]. Third, MDM proteins can promote p53 translocation out of the nucleus, thus preventing p53 binding to target genes [73]. The MDM N-terminal domain interactions with p53 constitute the primary interaction site: residues 18–26 within the p53 TAD forms a hydrophobic α-helix with key residues F19, W23 and L26 as key determinants, which facilitate binding to the MDM hydrophobic pocket (Figure 4) [71,72]. Specific residues within MDM2 and MDM4 critical for this interaction have been identified, including MDM2-L54 and MDM4-M53, both forming a hydrogen bond with p53-W23 [72,74]. Additional contact residues unique to the MDM4-p53 interaction were mapped to the acidic domain, which were physiologically relevant in a mouse model [57,58,75]. While secondary interaction sites for p53 have not been described in MDM2, it is known that the acidic domain can induce a conformational change in p53 through interactions with its N-terminal domain, resulting in inhibited p53 activity [76]. Collectively, these interactions form a complex regulatory network that significantly influences p53 function.

### Ubiquitination of p53

The most recognized activity of MDM proteins is the catalytic transfer and conjugation of ubiquitin molecules to p53. Although MDM4 lacks intrinsic E3 ubiquitin ligase activity, it enhances MDM2 ubiquitination activity within a heterodimer [77]. In MDM-mediated p53 ubiquitination, the outcome can lead to either protein degradation or nuclear export depending on MDM2 levels; low MDM2 levels cause p53 monoubiquitination, but high levels promote polyubiquitination [73]. One study challenged existing theories by suggesting that MDM2 homodimers only monoubiquitinate p53 independent of MDM2 levels using untagged MDM2 proteins [78]. The authors propose that earlier findings that linked ubiquitination type to MDM2 levels were due to the specific expression of GST-tagged MDM proteins [78]. However, no follow-up studies have been conducted to test this hypothesis. Key ubiquitination sites on p53 have been mapped to the six lysine residues located in the C-proximal regulatory region [79]. Mutation of these lysine residues reduces p53 degradation and enhances its transcriptional activity [79]. Additional ubiquitination sites within p53 N-terminal DNA-binding domain also contribute to its degradation, indicating the involvement of multiple residues in ubiquitination-associated degradation [80]. Interestingly, despite lacking E3 activity, MDM2-MDM4 heterodimerization incorporating MDM2 mutants with compromised E3 activity restores p53 ubiquitination [81]. This was further demonstrated using chimeric proteins generated by swapping the MDM2-RING with the MDM4-RING domains, alongside specific MDM mutations that restore E2 recruitment [63]. Such chimeric proteins harbor ubiquitination activity similar to wt MDM2, indicating functional E3 ubiquitin ligase activity [63].

### Regulation of *TP53* mRNA translation

**Figure 5:**
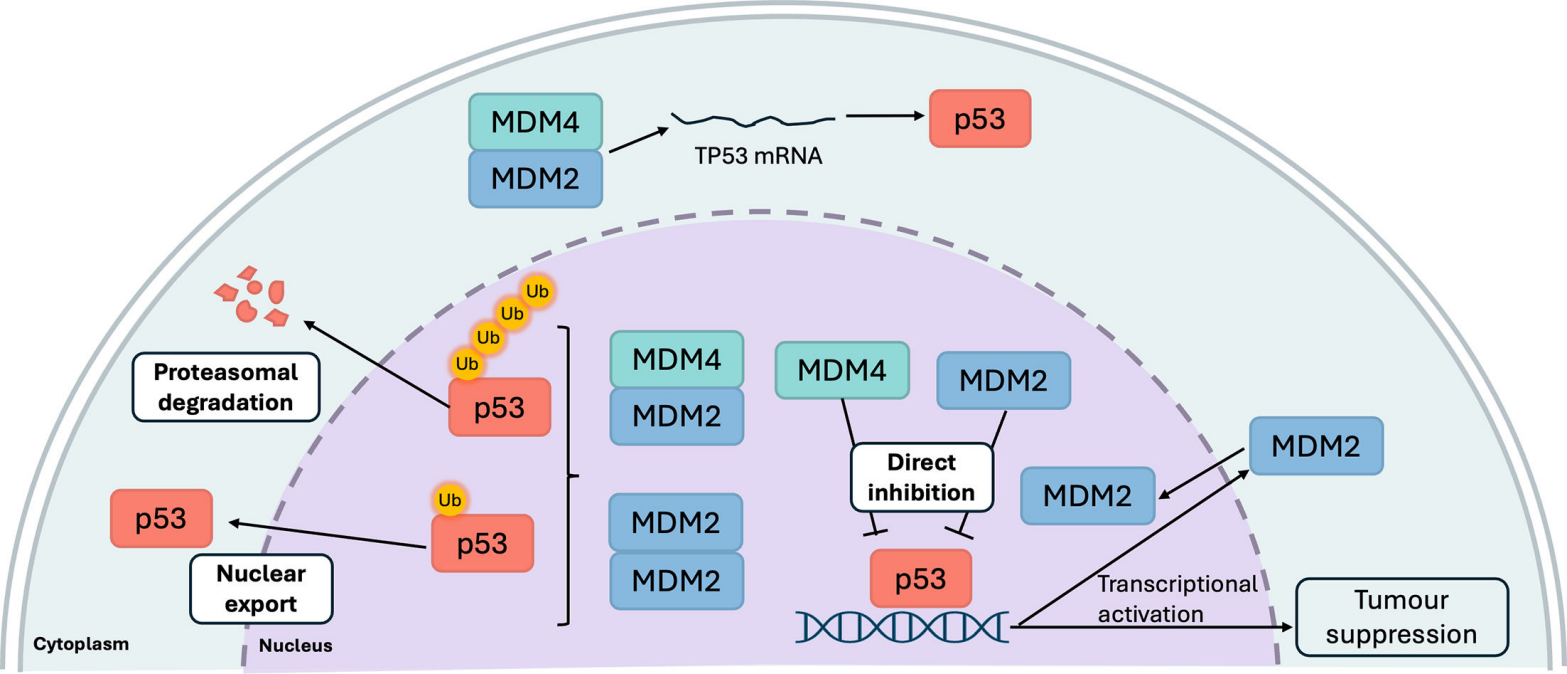
Mechanisms of p53 regulation by MDM2 and MDM4 proteins. MDM2 and MDM4 can directly bind and inhibit p53-mediated activation of tumor suppressor genes. MDM2 homodimers and MDM2-MDM4 heterodimers facilitate p53 proteasomal degradation and nuclear export depending on the type of ubiquitin linkage. Under genotoxic stress, MDM2 and MDM4 can act as internal ribosomal entry sites (IRES) trans-acting factors to promote p53 protein production by interaction with *TP53* mRNA. p53 activation can activate the transcriptional activation of MDM2 (created using BioRender.com). MDM2, mouse double-minute 2; MDM4, mouse double-minute 4.

Intriguingly, MDM2 has also been identified to harbor a positive effect on p53 levels by promoting *TP53* mRNA translation, thereby enhancing cellular apoptosis (Figure 5) [82]. This effect is dependent on ataxia-telangiectasia mutated ser/thr protein kinase (ATM)-mediated phosphorylation of MDM2 at S395 following genotoxic stress [82]. Phosphorylation at this site induced a conformation change in MDM2, facilitating its interaction with the methyl-CpG binding-encoding region of *TP53* mRNA via the RING domain [83–85]. This interaction triggers MDM2 SUMOylation and nucleoli localization [82]. Furthermore, the binding of MDM2 to *TP53* mRNA inhibits MDM2-mediated p53 polyubiquitination and degradation, while simultaneously stimulating *TP53* mRNA translation [82,84]. Additionally, ATM also mediates the phosphorylation of MDM4 at S403, allowing its binding to *TP53* mRNA, which subsequently alters the *TP53* mRNA secondary structure in favor of MDM2 binding [85]. Interestingly, phosphomimetic mutants of MDM2 (S395D) and MDM4 (S403D) form heterodimers with approximately three-fold higher affinity for each other, compared with the wt counterparts through N-terminal domain interactions [84]. This heterodimer retains the ability to ubiquitinate MDM2 and MDM4 but lacks activity against p53, presumably due to conformational change induced by phosphorylation [84]. This lack of p53 ubiquitination activity may also be partly attributed to p53-S15 phosphorylation by ATM, which confers protection from MDM2-mediated degradation [86]. Furthermore, MDM2 can also modulate *TP53* mRNA translation indirectly via regulation of the level of the ribosomal protein L26 [87]. L26 binds *TP53* mRNA and enhances its translation, while MDM2 can both directly inhibit L26 binding to *TP53* mRNA or mediate polyubiquitination and subsequent L26 degradation, thus preventing such interaction(s) under normal conditions [87]. MDM2 repression of L26 is relieved under genotoxic stress, allowing *TP53* mRNA translation [87]. Overall, these results indicate that ATM-mediated phosphorylation promotes MDM2 and MDM4 degradation while allowing p53 accumulation under genotoxic stress. This reveals a complicated regulatory network where MDM and p53 proteins modulate both their levels and activities.

## MDM substrate specificity

### IGF-1R

While the oncogenic activities of MDM proteins are primarily linked to their interaction with p53, the MDM proteins also play crucial roles in various cellular processes such as DNA synthesis, cell cycle control, differentiation, apoptosis and cell surface receptor signaling [3]. Furthermore, under *TP53*-null conditions, as seen in *TP53*-defective cancers, MDM proteins promote cancer cell survival and progression, underscoring their p53-independent role [3]. The IGF-1R is another protein frequently overexpressed in cancers and is involved in tumor cell transformation, growth and apoptosis prevention [37]. MDM2 interacts with the C-terminal region of IGF-1R within the β-subunit to facilitate its proteasomal degradation (Table 1) [37,47]. This regulation requires the presence of β-arrestin-1, which forms a complex with IGF-1R and binds to MDM2 between residues 161–400 [88]. Interestingly, IGF-1R can induce the expression of p53 and MDM2, while p53 can sequester MDM2 and inhibits the degradation of IGF-1R [37,89]. The competition between IGF-1R and p53 for MDM2 binding underscores the importance of understanding how inhibiting MDM2 to activate p53 affects IGF-1R tumor-promoting activities. Nutlin-3, a small molecule inhibitor that binds the MDM2 N-terminus, disrupts MDM2-p53 interactions and promotes IGF-1R ubiquitination and degradation [47,90]. However, Nutlin-3 treatment also activates IGF-1R downstream signaling through the canonical MAPK pathway, despite the induction of IGF-1R degradation [47]. This effect is potentially due to IGF-1R endocytosis induced by ubiquitination, which is known to enhance MAPK pathway activation [91].

### Androgen receptor

The androgen receptor (AR) plays a critical role in prostate cancer, acting as a major therapeutic target due to its altered activity that contributes to tumor initiation and progression. MDM2 is known to mediate the ubiquitination and degradation of AR, where MDM2 knockout results in increased AR expression in prostate cancer cells (Table 1) [38,92]. The specific interaction between MDM2 and AR has been mapped to the MDM2 C-proximal region (residues 341–491) containing the RING domain [38]. MDM2 and MDM4 are often overexpressed in prostate cancer cells; inhibition of MDM2 by Nutlin-3 activates p53 while reducing AR levels [93]. This decrease in AR levels might seem contradictory given the role of MDM2 in degrading AR. However, Nutlin-3 specifically disrupts the MDM2-p53 interaction while still allowing MDM2 to ubiquitinate other substrates such as IGF-1R and AR. This is supported by the observation that Nutlin-3 enhances AR ubiquitination, suggesting that Nutlin-3 redirects MDM2 from p53 inhibition to promoting AR degradation [94]. Interestingly, MDM2 expression coupled with low MDM4 levels stabilizes AR levels [93]. This finding indicates the need to include MDM4 in future studies on membrane receptor turnover and functional implications.

### Nbs1

MDM2 is also implicated in the DNA repair pathway in a p53-independent manner. The most studied PPI(s) in this context is between MDM2 and Nbs1 in modulating Mre11/Rad50/Nbs1 (MRN) DNA repair complex function (Table 1), where MDM2 overexpression has been shown to delay DNA repair [45]. The MRN complex plays an important role in double-stranded (ds) DNA break repair, where Nbs1 is required for ATM activation [95]. The MDM2-Nbs1 interaction has been mapped to residues 189–314 in MDM2 and 221–540 in Nbs1, which leads to co-localization of both proteins to DNA damage foci [45]. This inhibitory effect of MDM2 on dsDNA repair does not rely on its ubiquitin ligase activity and is likely due to a direct inhibitory effect [45]. MDM4 has also been implicated to associate with Nbs1 independent of MDM2, and this interaction has been detected at chromatin and is linked to increased genomic instability and transformation potential [96]. Interestingly, these effects appear to occur without affecting cell growth or survival. Aside from Nbs1, MDM2 interacts with and ubiquitinates other DNA repair proteins, such as Ku70, DNA polymerase eta and HBP1, further illustrating the multifaceted role of MDM2 in dsDNA repair pathways [97,99]. This highlights the extensive network of interactions mediated by MDM2.

### Other MDM substrates

Expanding on the aforementioned interactions, an assessment of MDM2 and MDM4 interacting partners and potential substrates using PPI databases, such as BioGRID (https://thebiogrid.org), IntAct (https://www.ebi.ac.uk/intact/home) and MINT (https://mint.bio.uniroma2.it), reveals a wide variety of associated proteins (Table 1) [100–102]. Comparison of the profile of MDM2- and MDM4-interacting proteins reveals both common and unique factors. Notably, the membrane protein E-cadherin (CDH1), which mediates homotypic cell–cell interactions and modulates epithelial–mesenchymal transition, a key feature of tumorigenesis–metastasis and cancer progression, is a substrate for MDM2 (Table 1) [44,103]. MDM2 is implicated in E-cadherin ubiquitination and down-regulation, thus promoting cancer cell migration and differentiation [44,104,105]. However, a database scan reveals a lack of MDM4 interactions with E-cadherin; it is feasible that such interaction may exist but has yet to be detected using current assays.

In addition to their unique interactors, MDM2 and MDM4 also share overlapping classes of interactors, including members of the p53 protein family, E2 ubiquitin-conjugating enzymes, E3 ubiquitin ligases and deubiquitinases such as USP2 [100]. Both MDM2 and MDM4 interact with polyubiquitin-C (a polyubiquitin precursor), suggesting a common ability to bind to the conserved ubiquitin fold. The interaction of MDM2 with the tumor suppressor and E3 family member, von Hippel-Lindau (Table 1), is notable in this context. Interestingly, both MDM proteins interact with a large number of ribosomal proteins, implying their roles in regulating protein synthesis. Additionally, MDM2 is also documented to interact with subunits of the 26S proteasome, highlighting its role in proteasomal degradation pathway, though less is known about MDM4 in this context.

Furthermore, a wide variety of protein kinases, both membrane-bound and soluble, have been documented as potential MDM2 substrates. Notable examples include IGF-1R, checkpoint kinase 2 and protein kinase Cβ (Table 1). Transcription factors are also key MDM2 targets with E2F1 documented to interact with both MDM proteins (Table 1). Furthermore, MDM2 interacts with a variety of GTPases and GTPase regulatory factors and thus potentially modulates a wide variety of signaling events. Interestingly, membrane proteins such as Notch1 are documented interactors, which merited further study in the context of vascular and neural disease processes. Overall, some key MDM-interacting partners have been highlighted here, but numerous additional interacting proteins exist, many of which have not been addressed extensively but play critical roles in various disease states and warrants further research.

## MDM-p53 regulation of endothelial function and angiogenesis

### Angiogenesis

Angiogenesis, the process of new blood vessel sprouting from pre-existing ones, plays an essential role in the growth and metastasis of tumors [106]. This process is facilitated by the endothelial cell monolayer that lines all blood vessels. Endothelial cells are activated by extracellular signals to regulate cellular growth, differentiation, migration and apoptosis [107]. Among the various downstream targets, vascular endothelial growth factor A (VEGF-A) is a key regulator of angiogenesis in both health and cancer [108]. In solid tumors, hypoxia (<1% O_2_) and lack of sufficient oxygenated blood serve as the major stress signals that promote tumor VEGF-A expression; secreted VEGF-A acts as a chemokine that stimulates angiogenesis by nearby blood vessels, leading to tumor neovascularization, growth and metastasis [109].

### VEGF-A

**Figure 6:**
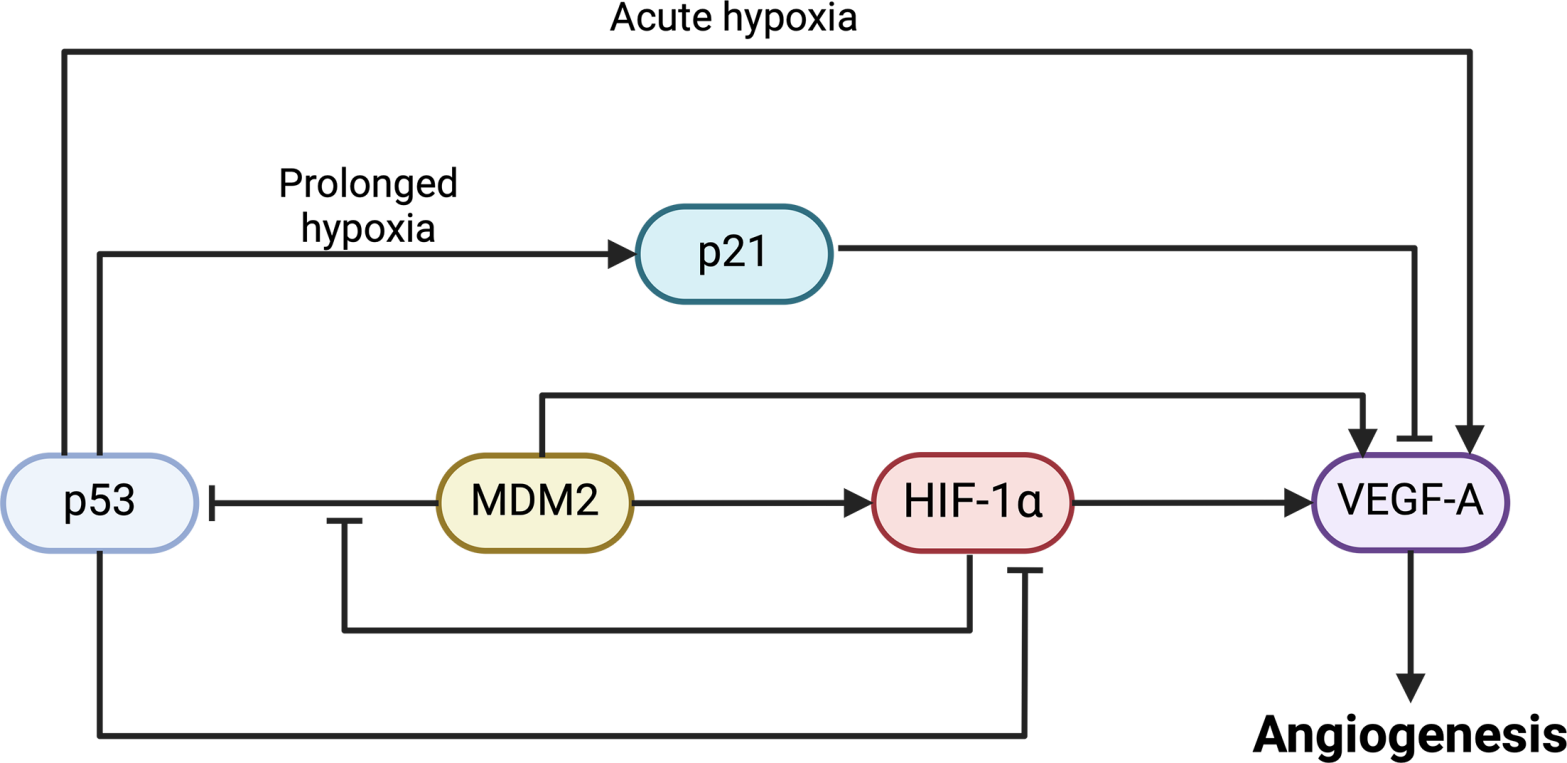
Interaction between MDM2 and p53 in hypoxia response. This schematic illustrates the regulatory network involving p53, MDM2 and downstream effectors during the cellular response to hypoxia. MDM2 modulates p53 stability and activity via ubiquitination. Hypoxia-inducible factor 1-alpha (HIF-1α) is stabilized under low oxygen conditions and can be up-regulated by MDM2 expression. HIF-1α suppresses MDM2-mediated inhibition of p53, thereby promoting p53 accumulation during hypoxia which in turn inhibits HIF-1α. HIF-1α promotes the expression of vascular endothelial growth factor A (VEGF-A), a key mediator in angiogenesis. Under acute hypoxia, p53 can directly increase VEGF-A levels, whereas prolonged hypoxic condition led to p53-mediated inhibition of VEGF-A via the activation of p21 (created using BioRender.com). MDM2, mouse double-minute 2.

There is a strong correlation between the MDM2 overexpression and increased VEGF-A levels in clinical cancer patient samples [110], implying a role for MDM2 in tumor angiogenesis. This observation is further supported by the finding that MDM2 inhibition also inhibits *VEGFA* expression with reduced angiogenesis (Figure 6) [108]. Further studies have identified the MDM2 RING domain as being responsible for binding to 3′UTR of *VEGFA* mRNA, increasing its stability and translation independent of p53 [111]. Apart from direct interaction with the *VEGFA* mRNA, MDM2 has also been found to regulate VEGF-A levels by up-regulating hypoxia-inducible factor-1α (HIF-1α) expression through binding to p53 [112,113]. HIF-1α is a subunit of HIF-1αβ heterodimer that is up-regulated in response to hypoxia and promotes *VEGFA* transcription by RNAPII [112]. Furthermore, other factors involved in VEGF-A up-regulation and promotion of angiogenesis, including THBS1, TNF-α and MMP9, are also up-regulated in response to MDM2 overexpression [114]. Together, these findings indicate the ability of MDM2 to regulate VEGF-A levels and activity via multiple independent pathways, indicating a key role of MDM2 in angiogenesis.

### HIF-1α

The angiogenic properties of MDM proteins can also be linked to their role as regulators of p53. Hypoxia induces the accumulation of p53 through HIF-1α-mediated suppression of MDM2, while activated p53 inhibits HIF-1α, forming a negative feedback loop [115,116]. Additionally, HIF-1α can block MDM2-mediated ubiquitination and degradation of p53, presumably by competing for the same binding site, thus forming a complicated regulatory loop [113]. While p53 is traditionally known as a negative regulator of angiogenesis, recent findings suggested a context-dependent effect of p53 on angiogenesis. Under acute hypoxia (0.5% O_2_, 4 h), p53 promotes angiogenesis by binding to the *VEGFA* promoter region and enhancing gene transcription in a HIF-1α-dependent manner [117]. In contrast, prolonged hypoxia (0.5% O_2_, 24 h) leads to p53-mediated activation of p21, which in turn inhibits *VEGFA* gene transcription [117]. Moreover, severe hypoxia (< 0.1% O_2_) triggers the expression of p53 regulated pro-apoptotic genes that inhibit anti-apoptotic AKT signaling, resulting in cell death [109].

### Tumor suppressor p53

MDM2 is known to suppress p53 activation and promote its degradation, thereby inhibiting p53-mediated suppression of angiogenesis. Mouse *MDM2* knockout-associated activation of p53 has been shown to increase the expression of pro-apoptotic genes *p21* and *PUMA* while decreasing the mRNA levels of pro-angiogenic factors *TIE2*, *VEGFA* and *KDR*, which encodes VEGF receptor 2 [118]. These results further translate into the reduction in angiogenesis through p53 activation in response to co-deletion of both MDM2 and MDM4 loci in mice [119]. Conversely, *TP53* loss has been shown to increase the VEGF-A levels and tumor neovascularization [120]. These findings underscore the critical role of MDM proteins in regulating p53-linked activity in angiogenesis. The frequent overexpression of MDM proteins combined with p53 down-regulation in tumors plays a key role in promoting angiogenesis under the prolonged hypoxic conditions induced by tumors. Thus, targeting the interaction between MDM proteins and HIF-1α could represent a promising target to inhibit tumor cell survival in hypoxic conditions.

Overall, the specific role of MDM proteins and p53 in response to hypoxia appears to be dependent on the severity and duration of hypoxia. For example, recent research indicates that inhibition of HIF-1α and p53 levels using siRNA does not prevent apoptosis in hypoxic conditions, which suggests the involvement of alternative pathways [121]. These findings point toward the need to build a comprehensive framework to address the discrepancies observed across different studies, which have likely arisen due to the use of different cell lines, varying oxygen concentration and differences in the duration of exposure. Given the substantial evidence supporting the role of MDM proteins in regulating the hypoxic responses and controlling angiogenesis, further research into its contribution to hypoxic response would be beneficial for targeted treatment development.

## MDM inhibitors

Given the significant role of MDM proteins in oncogenesis and p53 regulation, targeting MDM proteins to counteract these effects and selectively activate the p53 tumor suppressor has been under considerable interest for cancer treatment. MDM2, which is known for its direct role in p53 ubiquitination and degradation, is a primary target in this strategy [122]. The co-crystal structure of the N-terminal domain of MDM2 bound to p53 peptide containing the TAD revealed three major p53 residues involved in these interactions, which were used for the development of MDM2 inhibitors (Figure 4A; Table 2) [123].

**Table 2:** Summary of selected MDM2 and MDM4 inhibitors under clinical trials.

Type	Compounds	Nature	Disease	Combination	Clinical phase	Status	NCT number	Sponsor
MDM2 inhibitor	RG7112	Cis-imidazoline	Hematologic neoplasms	/	Phase 1	Completed	NCT00623870	Roche
	RG7388	Pyrrolidine	Relapsed or refractory acute myeloid leukemia	Cytarabine	Phase 3	Terminated	NCT02545283	Roche
			Relapsed or refractory acute leukemia/solid tumors	Venetoclax	Phase 1/2	Terminated	NCT04029688	Roche
	Navtemadlin	Piperidinones	Myelofibrosis	Best available therapy	Phase 2/3	Recruiting	NCT03662126	Kartos therapeutics
	Siremadlin	Pyrazolopyrrolidinone	Advanced solid and hematological tumors with wt *TP53*	Ancillary treatment	Phase 1	Completed	NCT02143635	Novartis
			Advanced solid tumors/lymphomas	/	Phase 1	Completed	NCT01877382	Daiichi sankyo
	Alrizomadlin	Spirooxindole	Metastatic melanomas/advanced solid tumors	Pembrolizumab	Phase 1/2	Recruiting	NCT03611868	Ascentage pharma
MDM2/MDM4 dual inhibitor	ALRN-6924	Peptide	Advanced/metastatic solid tumors	Paclitaxel	Phase 1	Active, not recruiting	NCT03725436	M.D. Anderson cancer center

Data are from https://ClinicalTrials.gov

wt, wild-type.

### Nutlin-3

**Figure 7:**
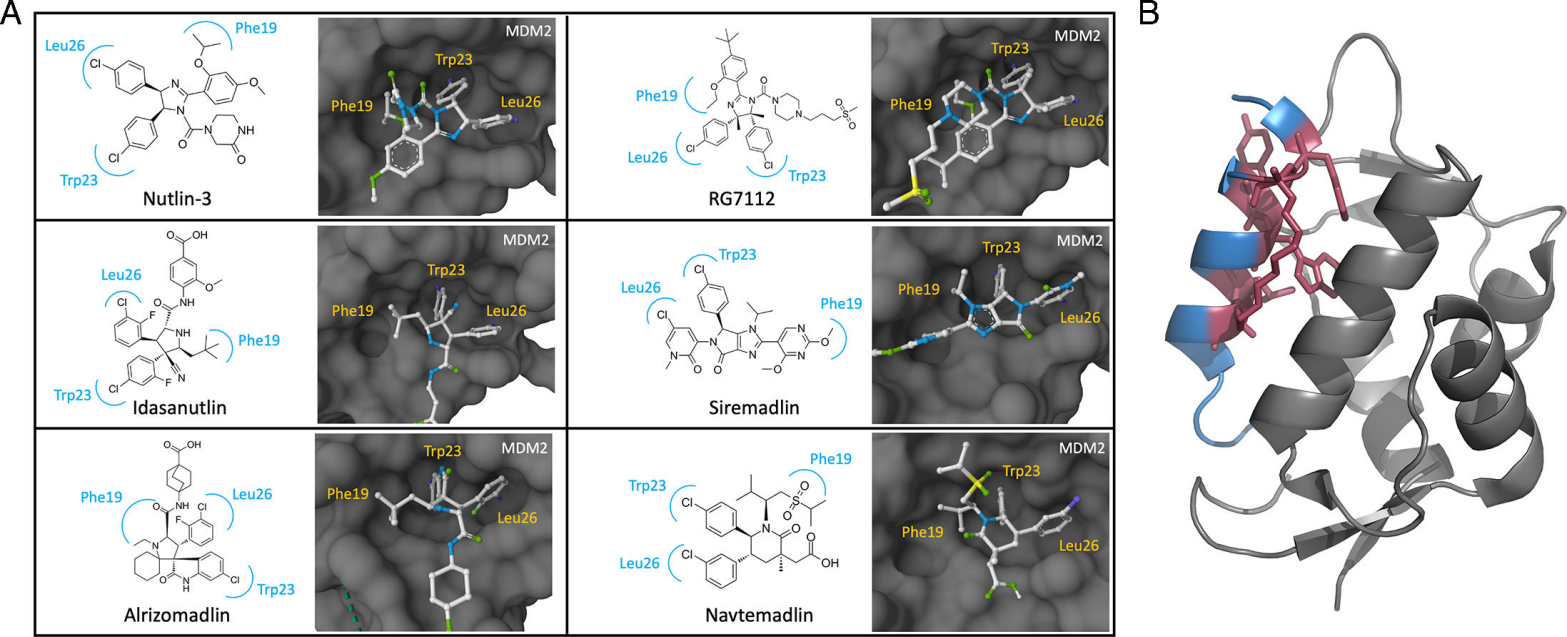
Binding of small molecule inhibitors and stapled peptides to MDM2 and MDM4. **(**A**)** Chemical and X-ray co-crystal structure of Nutlin-3 (PDB ID: 4J3E), idasanutlin (RG7388; PDB ID: 4JRG), siremadlin (HDM201; PDB ID: 5OC8), alrizomadlin (APG-115; PDB ID: 5TRF), Nnavtemadlin (AMG-232/KRT-232; Ppredicted using CB-DOCK2 [131]), bound to MDM2. MDM2 -binding pockets are labelled by p53 side chain in yellow. **(**B**)** X-ray co-crystal structure of ALRN-6924 (PDB ID: 8GJS) bound to MDM4. MDM4 is shown in grey and ALRN-6924 is shown in aquamarine. The hydrocarbon staple is shown as sticks in pink. Structures generated using PyMOL (www.pymol.org). MDM2, mouse double-minute 2; MDM4, mouse double-minute 4.

Nutlin-3 was the first MDM2 antagonist identified that binds to the p53-binding pocket of MDM2 (IC_50_~90 nM) and has shown preclinical efficacy by inducing cancer cell apoptosis and reducing tumor growth in mice [124,125]. Importantly, this effect is mediated through the activation of the p53 pathway in cancer cells harboring wt p53, whereas cells with mutated p53 were insensitive to the treatment [125]. Despite early success in preclinical studies, Nutlin-3 treatment has been shown to lead to the acquisition of *TP53* somatic mutations, rendering the treatment ineffective [126,127]. Interestingly, Nutlin-3 has been shown to induce cell cycle arrest in a *TP53*-mutant hepatocellular carcinoma cell line by p73 activation, though the same was not observed in other cell lines [126,128,129]. Furthermore, off-target effects and the rapid elimination of Nutlin-3 *in vivo* hinder its clinical application [70,130]. These findings emphasize the need for drug optimization and the use of combination therapies to achieve effective inhibition while reducing the development of resistance. Despite its limitations, Nutlin-3 has laid a strong foundation for the development of more effective inhibitors. Extensive research is currently being conducted on a wide range of Nutlin-3-derived compounds, with some progressing into clinical trials and showing promising therapeutic efficiency (Table 2) (Figure 7A).

### RG7112

RG7112, the first MDM2 inhibitor to progress into Phase 1 clinical trials, is a structurally modified analogue of Nutlin-3 with improved MDM2 affinity (IC_50_ ~18 nM), increased potency (*K_d_* ~11 nM) and enhanced selectivity with a 14-fold higher affinity for mutant compared with wt p53 [124,132,133] Preclinical evaluation of RG7112 in glioblastomas and osteosarcoma models demonstrates its effectiveness in triggering tumor cell apoptosis and reducing tumor volume, specifically in MDM2-overexpressing tumors with wt p53 [132,134]. In line with the preclinical evidence, Phase 1 trials of RG7112 demonstrated its effectiveness against leukemia (Table 2) [135]. Interestingly, a subset of the patients (2/19) harboring *TP53* mutations responded to the treatment, although continuous clinical improvement was not observed [135]. This observation suggests several possibilities such as heterogenous *TP53* clonal populations in patients, functional variants of *TP53* mutations or p53-independent activities of RG7112, which requires further investigation. Despite its anti-leukemia efficacy, the high dose requirement of RG7112 resulted in gastrointestinal and hematological toxicities [135]. A lower dosage reduces hematological side effects but also decreases treatment effectiveness in solid tumor patients, with 97% of the patients still experiencing adverse effects [22]. The relatively severe hematological and gastrointestinal toxicities at the required high doses of RG7112 hinder its progression beyond Phase 1 trials. Nevertheless, these studies underscore the need for developing inhibitors that are both more potent and less toxic.

### RG7388

Despite the absence of follow-up studies for RG7112, its research laid the groundwork for the discovery of idasanutlin (RG7338). Idasanutlin possesses a pyrrolidine scaffold with increased potency (*K_d_* ~0.15 nM) to address the limitations associated with RG7112 [136,137]. Additionally, idasanutlin showed improved binding affinity for MDM2 (IC50 ~6 nM) and enhanced selectivity (344-fold), enabling the adaptation of a lower dosage regimen [136,137]. Idasanutlin has frequently been studied in combination with other drugs. The most advanced study is the MIRROS phase 3 clinical trial (NCT02545283; Table 2), where RG7338 was evaluated in combination with cytarabine for the treatment of acute myeloid leukemia (AML). Although initial Phase 1/1b data demonstrated the efficacy and tolerability of idasanutlin both alone and in combination therapy, subsequent Phase 3 trial indicated that the addition of idasanutlin did not improve the overall survival or complement remission rate in AML patients [138]. In addition, despite the implementation of a lower dose regimen, gastrointestinal and hematological associated adverse effects were still evident across disease type, leading to frequent abrogation of the clinical trials [138,139]. Currently, research is exploring idasanutlin in new combinations, such as with chemotherapy or venetoclax, for relapsed or refractory leukemia and solid tumors (NCT04029688; Table 2).

### Navtemadlin

Navtemadlin (AMG-232/KRT-232) is a second-generation MDM2 inhibitor with a piperidinone scaffold, with remarkable MDM2-binding affinity (IC_50_ ~0.6 nM; *K_d_* ~0.045 nM) [140]. The first clinical trial of navtemadlin showed good pharmacokinetics profiles with 66% of the solid tumor patients achieving stable disease [141]. Based on this success, multiple clinical trials are underway, including two Phase 3 trials. One of these is assessing the use of navtemadlin as treatment in myelofibrosis (MF) patients given the overexpression of MDM2 on CD34+ patient cells (NCT03662126, Table 2) [142]. Preliminary evidence revealed that an 87% reduction in CD34+ cells in peripheral blood and decreased variant allele frequency in key genes were seen in some MF patients, suggesting a potential disease-modifying effect of navtemadlin, warranting further studies [143]. These results also suggest potential use of other MDM2 inhibitors for MF therapy.

### Siremadlin

Evaluation of siremadlin (HDM201), a next-generation MDM2 inhibitor based on a pyrazolopyrrolidinone scaffold, has demonstrated the effectiveness of an intermittent high-dose regimen (Figure 7A) [144]. This approach not only mitigated p21-induced cell cycle arrest but also promoted a more robust anti-tumor activity through increased activation of p53, leading to the activation of p53-up-regulated modulator of apoptosis, a pro-apoptotic protein [144]. This dosing regimen is also thought to provide a period for hematological recovery in patients between doses to reduce off-target patient toxicity by MDM2 inhibitors [144]. Phase 1 trials on cancer patients with solid tumors and acute leukemias showed promising efficiency especially in AML patients, although hematological toxicities were still evident (Table 2) [145]. Furthermore, a study on milademetan (RAIN-32/DS3032b) comparing continuous and intermittent dosing in sarcoma patients indicated a lower incidence of severe thrombocytopenia with intermittent dosing (15% vs 35%) (Table 2) [145,146]. These findings provided basis for the application of intermittent dosing regimen for MDM2 inhibitors, which has been incorporated into the majority of the latest clinical developments.

### Alrizomadlin

While MDM2 inhibitors are often evaluated in combination with cytotoxic drugs, alrizomadlin (APG-115) is being studied with immune checkpoint inhibitors targeting PD-1 (Figure 7A). Pre-clinical study showed that treatment with a combination of alrizomadlin and anti-PD-1 in mice was able to elicit anti-tumor activity with increased efficiency than anti-PD-1 alone and is irrespective of *TP53* mutation status [147]. This is thought to be due to the activation of p53 by alrizomadlin, which stimulated transition of proliferation-promoting M2 to proliferation-inhibiting M1 macrophages in the tumor microenvironment [147]. Additionally, the increased efficiency of the combination therapy is also attributed to alrizomadlin-induced PD-L1 expression on tumor cells [147]. These findings are being further evaluated under a Phase 2 trial to confirm the findings in human, which showed promising preliminary efficacy in multiple tumors (NCT03611868; Table 2) [148]. These findings provided new opportunity to overcome the limitation of MDM2 inhibitors that are only active against wt p53, which represents only 50% of the cancer patients [8].

### ALRN-6924

The involvement of MDM4 in p53 inhibition and degradation combined with frequent MDM4 overexpression in cancer interferes with p53 activation by MDM2 inhibitors [4,149,150]. Therefore, dual targeting of both MDM2 and MDM4 is favorable for full p53 rescue and activation in disease states. However, current MDM2 inhibitors often lack activity against MDM4 due to its distinct p53-binding pocket structure influenced by M53 and Y99 residues, whereas novel MDM4 inhibitors suffer from potency and selectivity issues [151]. Among the ongoing attempts to discover MDM4 inhibitors, ALRN-6924, an α-helical stapled peptide, that acts as dual MDM2/MDM4 inhibitor demonstrated high efficacy against multiple cancer cell lines possessing wt p53 (Figure 7B) [152]. The first human trial of ALRN-6924 confirmed its anti-tumor activity with mild side effects, particularly a lower myelosuppressive effect was observed compared with the MDM2 inhibitors [153]. Currently, a Phase 1b trial of ALRN-6924 combined with paclitaxel for treating advanced metastatic tumor is ongoing (NCT03725436, Table 2).

### Limitations and anti-cancer efficacy

The exploration of MDM inhibitors has emerged as a promising avenue for cancer therapy, with numerous candidates advancing into clinical trials. However, many issues remain to be addressed, one of which is the gastrointestinal and hematological toxicities that are dose-limiting. These toxicities have likely resulted from p53 activation in normal tissues, causing disruption in cell cycle control or apoptosis of healthy cells. Specifically, hematological toxicity is attributed to MDM2 inhibition in the bone marrow, which disrupts normal hematopoiesis by inducing p53 activation that results in the subsequent death of hematopoietic stem cells and affecting megakaryocyte differentiation that is responsible for platelet production [154,155]. However, one study with the MDM2 inhibitor MI-219, which was discontinued due to low potency, indicated that while p53 activation occurs in both healthy and cancer cells, the effect is milder in normal cells, leading only to cell cycle arrest compared with apoptosis in tumor cells [156]. This finding suggests the potential for developing more potent and selective inhibitors that could minimize adverse events through reduced dosages or innovative delivery methods. Recently, the proteolysis-targeting chimera (PROTACs) MDM2 degrader MD-265 showed promising activity in enhancing survival rates in mice with leukemia carrying wt p53. MD-265 effectively degraded MDM2 with no significant signs of toxicity, presenting a new avenue for the development of MDM2-targeting drugs [157]. Furthermore, resistance to MDM inhibitors, through p53 mutations or up-regulation of anti-apoptotic genes, and MDM4 presents additional challenges [126,158]. Current research into combination therapies might reduce resistance development in patients, but long-term study would be required to confirm this effect and examine how resistance development might affect the treatment efficiency in patients.

## Conclusions

Considerable research has focused on the role of MDM2 and MDM4 as negative regulators for p53, especially on their ubiquitination activity that facilitates the degradation of p53. While these two proteins share sequence and structural similarities, their functional differences mark the understanding of their interactions a key area of study. Structural analyses have shed light onto the distinct activities of various MDM oligomers, though inconsistent results have been reported across studies. Furthermore, clinical studies of MDM mutations highlight the complex relationship between mutations and disease outcomes, where contradictory results suggest that no single mutation directly correlates with disease risk. In addition, most studies focused solely on one of the proteins; a more complete understanding of the role of MDM proteins in disease states would require an integrated approach to incorporate both proteins into studies.

MDM proteins are often overexpressed or mutated in cancers, causing dysregulation in their activity. Although these alterations were initially thought to occur independently of p53 mutations, recent studies reveal that both events can co-occur, suggesting a broader, p53-independent, oncogenic role of the MDM proteins. Indeed, increasing evidence has focused on the involvement of MDM proteins in various p53-independent cellular signaling pathways, including angiogenesis, that contribute to tumorigenesis. However, the contribution of MDM4 to such regulatory events remains relatively unknown. Further study on these p53-independent pathways could inspire the development of new therapeutics against cancers.

## Abbreviations

AML: acute myeloid leukemia
AR: androgen receptor
FL: full-length
IGF-1R: insulin-like growth factor 1 receptor
MDM2: mouse double-minute 2
MDM4: mouse double-minute 4
MRN: Mre11/Rad50/Nbs1
NES: nuclear export signal
NLS: nuclear localization signal
RING: Really Interesting New Gene domain
SNP: small nucleotide polymorphism
VEGF-A: vascular endothelial growth factor A
WT: wildtype
